# Mechanical pressure on endothelial cells mediates remote ischaemic preconditioning‐induced neuroprotection via miR‐126

**DOI:** 10.1113/EP093541

**Published:** 2026-09-06

**Authors:** Xiaojie Wang, Lei Yan, Hongwei Zhu, Yifan Li, Qi Liu, Wei Li, Kerui Gong, Zhijun Zhao, Chunyang Zhang, Gang Fu, Guo Shao, Xunming Ji

**Affiliations:** ^1^ Beijing Key Laboratory of Hypoxic Conditioning Translational Medicine Xuanwu Hospital Capital Medical University Beijing PR China; ^2^ Inner Mongolia Key Laboratory of Hypoxic Translational Medicine Baotou Medical college Baotou PR China; ^3^ Center for Translational Medicine The Third People's Hospital of Longgang Clinical Institute of Shantou University Medical college (The Third People's Hospital of Longgang District Shenzhen) Shenzhen PR China; ^4^ Ultrasound Imaging Department The Second Affiliated Hospital of Baotou Medical College Baotou PR China; ^5^ Department of Oral and Maxillofacial Surgery University of California San Francisco San Francisco California USA; ^6^ Department of Neurosurgery The First Affiliated Hospital of Baotou Medical College Baotou PR China

**Keywords:** endothelial cells, higher pressure, miR‐126, neuroprotection, remote ischaemic preconditioning

## Abstract

Remote ischaemic preconditioning (RIPC) uses brief limb ischaemia to protect distant organs, but the mechanism of travel of the protective signal remains unclear. This study investigated whether endothelial cells (ECs) contribute to RIPC‐induced neuroprotection and the underlying mechanisms. Ten healthy volunteers (5 men, 5 women) underwent RIPC. Blood velocity was measured by Doppler ultrasound, and vascular wall pressure (VWP) was computed from 3D fluid‐structure interaction models based on magnetic resonance imaging data. Human microvascular endothelial cells (HMEC‐1) were exposed to cyclic higher pressure (CHP) for five cycles. miR‐126 expression and promoter DNA methylation were assessed by real‐time PCR and bisulfite sequencing. DNA methyltransferases (DNMTs) and global methylation levels were measured. SH‐SY5Y neurons were incubated with exosomes from HMEC‐1 culture medium and then subjected to oxygen–glucose deprivation/reperfusion (OGD/R). Neuronal viability and apoptosis were evaluated by MTS assay and flow cytometry, and damage by spectrin and cleaved caspase‐3 levels. Dicrotic waves were induced in 9 of the 10 participants following RIPC treatment. VWP transiently increased following RIPC. CHP reduced the expression and activity of DNMTs and induced the hypomethylation of the miR‐126 promoter sequence in HMEC‐1 cells, leading to an increase in miR‐126 levels. Exosomes from CHP‐treated HMEC‐1 cells decreased SH‐SY5Y cell injury under OGD/R. RIPC transiently increased VWP in healthy volunteers. The results of this study indicate that CHP treatment may induce the production of neuroprotective molecules by ECs, which may protect against ischaemia/hypoxia‐induced neuronal cell injury in vitro, possibly through a CHP‐induced effect involving miR‐126.

## Abstract (highlights)

- **What is the central question of this study?**
  Do endothelial cells sense changes in mechanical pressure induced by remote ischaemic preconditioning (RIPC) and if so does this contribute to neuroprotection via miR‐126?
- **What is the main finding and its importance?**
  RIPC can transiently increase the vascular wall pressure and induce a dicrotic wave in humans. Endothelial cells treated with cyclic higher pressure (CHP) showed decreased expression and activity of DNA methyltransferases and increased miR‐126 expression along with its promoter hypomethylation. Exosomes from endothelial cells treated with CHP protected neurons from oxygen–glucose deprivation/reperfusion‐induced injury. These findings suggest that mechanical force may act as a RIPC trigger and identify CHP‐induced endothelial miR‐126 as a novel neuroprotective mechanism.

## 1 INTRODUCTION

Remote ischaemic preconditioning (RIPC) uses brief ischaemic episodes at a distant site to protect organs such as the brain, heart and kidneys from severe ischaemia. Several studies have presented evidence of the potential of RIPC in reducing organ damage, improving recovery and enhancing tissue repair mechanisms, thereby positioning it as an effective, non‐invasive therapeutic approach (Anttila et al., 2023; Jia et al., 2024; Liu et al., 2024). RIPC confers neuroprotection against brain injury through certain molecular mechanisms (such as metabolic reprogramming and protein modulation), illustrating that pathway modulation represents a viable neuroprotective strategy for neurological conditions involving ischaemia or hypoxia (England et al., 2017; Han et al., 2024). Although the exact mechanisms through which RIPC limits ischaemic injury in distant organs remain unclear, the release of autacoids (protective molecules) that increase the ischaemia/hypoxia tolerance of cells in distant organs is currently an accepted hypothesis (Keevil et al., 2024). Endothelial cells (ECs) in the ischaemic region may play a crucial role in mediating the neuroprotective effects of RIPC by releasing factors such as nitric oxide (NO) (Mollet et al., 2024).

ECs line the insides of all blood vessel walls in a monolayer and serve as an active and selective barrier between blood and surrounding interstitial tissue (Jaffe, 1985). Owing to their location and structure, vascular ECs experience biomechanical forces, including pressure, stretch and shear stress, that vary with time (Katoh, 2023). These biomechanical forces can affect the function of ECs through various signalling pathways (Li et al., 2005). The mechanical environment can modulate the endothelial Akt and endothelial nitric oxide synthase (eNOS) signalling pathways, which are critical for cell survival and protection (Peng et al., 2003). In vivo studies have shown that enhanced perfusion pulsatility alone increases coronary blood flow via both the NO and the KCa channel‐dependent pathways (Paolocci et al., 2001). Shin et al. (2002) demonstrated that ECs are capable of detecting and reacting to physiological levels of cyclic pressure, thereby adjusting cell proliferation and apoptosis in a pressure‐specific manner. Culture medium derived from hypoxia‐conditioned ECs was found to protect human intestinal cells against hypoxia–reoxygenation‐induced injury. This suggested that ECs may play a role in this protection mechanism by releasing specific molecules induced by recurrent hypoxic episodes (Hummitzsch et al., 2014). These molecules can be packaged into exosomes to relay protective information from ECs (Davidson et al., 2018). RIPC can be initiated by temporarily blocking blood flow to a limb using a blood pressure cuff inflated to 180 mmHg or higher. ECs exposed to cyclic pressure may undergo changes in gene expression during this process owing to the blood pressure stimulation (Anwar et al., 2012). However, it is not yet known whether ECs release protective molecules after being subjected to cyclic biomechanical forces. The responsiveness of ECs to mechanical stimuli suggests that forces, such as pressure, can modulate the expression of regulatory microRNAs (miRNAs), which may subsequently mediate remote intercellular communication and confer neuroprotective effects (Neth et al., 2013).

miRNA‐126, an EC‐specific miRNA (Shao et al., 2025), is regarded as one of the key miRNAs in preserving vascular integrity and has a significant involvement in neuroprotection following cerebral ischaemia–reperfusion (Xiao et al., 2020). There is a close association between miR‐126 expression and human essential hypertension (Kontaraki et al., 2014; Liu et al., 2018). Preliminary studies by Liu et al. indicated an increase in miR‑126 expression in patients with hypertension (Liu et al., 2018). We previously reported that RIPC can increase serum exosomal miR‐126 levels (Cui et al., 2020). The aim of the present study was to investigate whether the RIPC‐induced cyclic elevation of pressure contributes to neuroprotection by increasing miR‐126 expression.

## 2 Methods

### 2.1 Ethical approval

Ethical approval for this study was obtained from the Research Ethics Committee of Baotou Medical College (approval no. BYLL2021‐011). This study conformed to the standards set by the 1964 *Declaration of Helsinki* and its later amendments, except for registration in a database. Written informed consent was obtained from all participants.

### 2.2 Participants

A total of 10 healthy undergraduate college students (5 men and 5 women, aged 20–30 years) were recruited between October 2021 and December 2021 from Baotou Medical College. All participants were taking no medications, had no previous medical history, and volunteered to participate in the present study. All participants had normal blood pressure (mean systolic, 116.7 ± 3.2 mmHg; mean diastolic, 69.8 ± 2.4 mmHg). Each participant served as their own control, and their pre‐RIPC measurements were used as baseline references to evaluate the effects of RIPC.

### 2.3 RIPC procedures

The RIPC protocol involved subjecting the participants to five cycles of simultaneous bilateral arm ischaemia lasting 5 min each, followed by 5 min of reperfusion. These procedures were performed using identical electric auto‐control devices (Cui et al., 2020). During the ischaemic phase, the cuff pressure was set to 220 mmHg and reduced to 0 mmHg during reperfusion. Although lower pressures (20 mm greater than systolic blood pressure) may be sufficient to induce limb ischaemia (Randhawa et al., 2015), a pressure of 220 mmHg was used to ensure complete and rapid occlusion in all participants, which is consistent with protocols employed in other studies examining endothelial responses to RIPC (Bannell et al., 2023; Maxwell et al., 2019; Seeger et al., 2015).

### 2.4 Blood velocity measured by Doppler ultrasound monitor

Doppler ultrasound is highly reliable for measuring both the mean and maximum blood flow velocities in peripheral arteries (Guirro et al., 2017). In this study, the ultrasonographer used a Siemens Acuson ×300 Premium Edition diagnostic ultrasound machine (Mountain View, CA, USA), equipped with a 9–12‐MHz multi‐frequency broadband linear transducer, to measure blood velocity in the right internal carotid artery (ICA). The intra‐class correlation coefficients for these measurements were 0.501–0.866, with a standard error of measurement of 0.81–9.45 cm/s (Guirro et al., 2017). The ultrasound machine was calibrated before commencement of the study. Various parameters were measured at the beginning of RIPC and at minute 3 of each cycle of cuff inflation and deflation during RIPC. These parameters included the systolic peak, end‐diastolic velocity, maximum and average speed in the cardiac cycle (TAMAX and TAMEAN, respectively), pulsatility index, resistance index, systole/diastole ratio and body flow volume.

### 2.5 Magnetic resonance imaging data acquisition and analysis

3D in vivo magnetic resonance images of the internal carotid artery (ICA) were acquired from volunteers at The Second Affiliated Hospital of Baotou Medical College using a SIGNA™ Architect 3.0T scanner (Signa Horizon EchoSpeed, GE Healthcare, Chicago, IL, USA). Various imaging sequences, including cine phase contrast (CINE PC), T1‐weighted imaging (T1WI), T2WI and time‐of‐flight magnetic resonance angiography (TOF‐MRA), were used to obtain cervical arterial blood flow information. All images were obtained using the following parameters: CINE PC (flip angle = 20°; field of view = 160 × 160 mm; matrix = 256 × 256; velocity encoding = 90; slice thickness = 4 mm; 1 axial slice; trigger type = cardiac gating), T1WI (flip angle = 110°; field of view = 160 × 160 mm; matrix = 320 × 320; repetition time = 724 ms; echo time = 12.5 ms; slice thickness = 2 mm; 15 axial slices), T2WI (flip angle = 142°; field of view = 160 × 160 mm; matrix = 320 × 320; repetition time = 2,263 ms; echo time = 130.4 ms, slice thickness = 2 mm; 15 axial slices), TOF‐MRA (flip angle = 20°; field of view = 160 × 160 mm; matrix = 360 × 360; repetition time = 22 ms; echo time = 2.8 ms; slice thickness = 1.2 mm; 47 axial slices). MRI data analysis was performed by Jingke Technology Co., Ltd. (Shenzhen, China) to calculate the vascular wall pressure (VWP).

### 2.6 Cell culture

Human microvascular ECs (HMEC‐1) were purchased from the Chinese Academy of Medical Sciences and the Peking Union Medical College and cultured in M199 endothelial growth medium supplemented with 10% fetal bovine serum (FBS), endothelial cell growth supplement (4 µL/mL), epidermal growth factor (0.1 ng/mL), basic fibroblast growth factor (1 ng/mL), heparin (90 µg/mL) and hydrocortisone (1 µg/mL) (Reiss et al., 2004). Human neuroblastoma SH‐SY5Y cells were obtained from the Cell Bank of the Chinese Academy of Sciences (cat. no. SCSP‐5012; American Type Culture Collection, cat. no. CRL‐2266). The short tandem repeat (STR) profile of SH‐SY5Y cells in this study showed a 100.0% match with the profile listed for SH‐SY5Y cells in Cellosaurus (https://web.expasy.org/cellosaurus/CVCL_0019), confirming the authenticity of the cell line. SH‐SY5Y cells were cultured in RPMI 1640 medium supplemented with 10% FBS. For both the M199 and RPMI 1640 media, 100 U/mL of penicillin/streptomycin was added during cell culture.

### 2.7 Cyclic pressure set‐up

HMEC‐1 cells were divided into two groups: control and cyclic higher pressure (CHP) groups. Cells in the control group were cultured under static atmospheric pressure conditions without any additional pressure stimulation. In contrast, cells in the CHP group were cultured in a cyclic pressure chamber. The cyclic pressure system used in this study was designed to expose the cultured HMEC‐1 cells to pressure regimes suitable for transient and repeated pressure changes. During experiments, HMEC‐1 cells were placed in a sealed and humidified pressure chamber (Naturethink, Shanghai, China) and exposed to repeated pressure episodes (five cycles of 5 min each) defined by a range of approximately 100–0 mmHg above atmospheric pressure (Tworkoski et al., 2018). The pressure chamber was calibrated before each experiment using a digital manometer (precision ±1 mmHg). Pressure was applied using a controlled inflow of a gas mixture (74% N_2_ + 21% O_2_ + 5% CO_2_), and the pressure waveform was continuously monitored using an inline pressure sensor. Control cells were placed in an identical chamber and maintained at ambient atmospheric pressure without cyclic pressurization. All experiments were performed in a humidified incubator at 37°C with 5% CO_2_. The in vitro application of realistic mechanical pulsatile forces can critically inform mechano‐signalling studies (Peng et al., 2000). Following the cyclic pressure treatment, HMEC‐1 cells were cultured for 24 h. Subsequently, HMEC‐1 cells and culture medium were collected separately.

### 2.8 Bioinformatic analysis of differential expression of exosomal miRNA in culture medium

The differential expression of exosomal miRNA was detected via high‐throughput sequencing (HTS) using an Illumina instrument at Lianchuan Bio Co. Ltd. The DESeq2 method was used for differential miRNA expression analysis (Love et al., 2014). A corrected *P*‐value, known as the *Q*‐value, of <0.05 was considered statistically significant for identifying differentially expressed miRNAs.

### 2.9 Real‐time PCR detection for miR‐126 and DNA methyltransferases

The levels of miR‐126 in exosomes and HMEC‐1 cells were determined using RT‐qPCR following established protocols (Cui et al., 2020). Briefly, RNA was extracted from exosomes and cultured cells using the Tissue and Cell Total RNA Extraction kit (GenePharma, Shanghai, China), followed by reverse transcription using GenePharma's Hairpin‐it™ kit. U6 snRNA was used as the internal reference for miRNA‐126. DNA methyltransferase (DNMT) levels in HMEC‐1 cells were measured using RT‐qPCR. Total RNA was isolated using Trizol reagent (Thermo Fisher Scientific, Waltham, MA, USA), followed by reverse transcription using a Superscript III FIRST Strand synthesis kit (Thermo Fisher Scientific, Waltham, MA, USA). β‐Actin was used as the internal control. The stability of U6 and β‐actin across all experimental conditions was confirmed using the coefficient of variation method (CV < 0.05), and CHP treatment was found to have no significant effect on their expression. RT‐qPCR was performed using three independent biological replicates (i.e., separate culture cell or culture medium batches), with each sample measured in technical triplicate using an ABI 7900 Real‐Time PCR System (Thermo Fisher Scientific). Data analysis was conducted using the 2^‐ΔΔ^ *^C^*_T_ method as previously described (Cui et al., 2020). Primer sequences are listed in Appendix Table A1.

### 2.10 Bisulfite‐modified DNA sequencing

To investigate the impact of pressure on miR‐126 promoter methylation, the CpG island promoter methylation status was assessed in HMEC‐1 cells using bisulfite‐modified DNA sequencing (BMDS) (Andersen et al., 2015). Briefly, genomic DNA samples from HMEC‐1 cells were treated with sodium bisulfite. An online program (http://www.urogene.org/cgi‐bin/methprimer/methprimer.cgi) was used to predict a CpG island, and MethPrimer was subsequently used to design primers targeting miR‐126 genes. The modified DNA was subsequently amplified using nested PCR primers (Appendix Table A2) that were specifically tailored to the modified strand of the miR‐126 promoter. The PCR products were subjected to gel purification. Subsequently, the purified PCR products were cloned into the PCR2.1 vector (Thermo Fisher Scientific). The vectors were introduced into DH5α competent bacterial cells. Recombinant plasmid DNA was isolated from a minimum of 10 positive clones for each PCR product and subjected to DNA sequence analysis at Sangon Biotech (Shanghai,China).

### 2.11 DNMT activity assay

Nuclear proteins from HMEC‐1 cells were cultured under normal conditions or treated with CHP and then purified using a EpiQuik Nuclear Extraction Kit II (Epigentek, Farmingdale, NY, USA). Total DNMT activities for total DNMT, DNMT1 and DNMT3B were measured using a DNMT Activity Assay Kit (Epigentek) according to the manufacturer's instructions. DNMT activity was assessed in three independent biological replicates (i.e., separate cell cultures), with each sample measured in triplicate.

### 2.12 DNA global methylation

Repeated DNA elements, such as Alu and long interspersed nuclear element‐1 (LINE‐1), are frequently used as proxies for quantifying global DNA methylation levels. Genomic DNA was extracted from ECs using the QIAamp DNA Mini Kit (Qiagen DNA extraction kit, Qiagen Inc. Hilden, Germany) and subjected to bisulfite treatment using an EZ DNA Methylation kit (Zymo Research, Irvine, CA, USA). To assess the methylation levels of Alu and LINE‐1 sequences, a technique known as combined bisulfite restriction analysis (COBRA) was used. Following bisulfite treatment, the modified DNA was subjected to PCR amplification using the Alu and LINE‐1 primers (Appendix Table A3). The PCR products derived from the Alu sequences were digested with 10 U of *Mbo*I, and those from LINE‐1 were digested with 10 U of either *Taq*I or TSP509I. The digested PCR products were separated using 3% agarose gel electrophoresis. All experiments were performed with four independent biological replicates.

### 2.13 Western blotting

The cells were lysed using RIPA buffer (Beyotime Biotechnology, Shanghai, China), and protein levels of DNMT1, DNMT3A, DNMT3B, spectrin, caspase‐3 and β‐actin were determined using western blotting. Primary antibodies against DNMTs were obtained from Novus Biologicals (Littleton, CO, USA); antibodies against spectrin, caspase‐3 and β‐actin obtained were from Cell Signaling Technology (Danvers, MA, USA), as previously reported (Zhang et al., 2023). Protein lysates (20 µg per lane) were separated on 10% SDS‐PAGE gels and transferred onto polyvinylidene difluoride membranes (Roche Diagnostics, Indianapolis, IN, USA). Membranes were blocked with 5 % non‑fat milk in Tris‐buffered saline with Tween‐20 for 1 h at 20°C. Primary antibodies were used at the following dilutions: anti‑DNMT1 (1:1000), anti‑DNMT3A (1:800), anti‑DNMT3B (1:800), anti‑spectrin (1:1000), anti‑cleaved caspase‑3 (1:500) and anti‑β‑actin (1:5000). Horseradish peroxidase‑conjugated secondary antibodies were used at a 1:5000 dilution. Blots were developed using ECL reagent and imaged using a ChemiDoc system (Bio‐Rad Laboratories, Hercules, CA, USA). All western blots were performed in three independent biological replicates.

### 2.14 Incubation of SY5Y cells with exosomes from CHP‐treated HMEC‐1‐conditioned media

The medium obtained from HMEC‐1 cells cultured under normal conditions or treated with CHP was collected and filtered through a sterile filter. Exosomes were isolated from the medium using the Minute™ High‐Efficiency Exosome Precipitation Reagent (Invent Biotechnologies, Beijing, China). Briefly, cell culture supernatants were collected and centrifuged to remove debris. The supernatant was mixed with the precipitation reagent and incubated at 4°C overnight. Exosomes were pelleted by centrifugation and resuspended in phosphate‐buffered saline. Exosome purity and integrity were verified using electron microscopy and nanoparticle tracking analysis and the presence of exosomal markers (CD63, HSP70 and TSG101) was verified via western blotting. SH‐SY5Y cells were incubated with exosomes following a previously described method. Overall, three groups were established for subsequent experiments following a 24‐h incubation period (Cui et al., 2020). SH‐SY5Y cells were cultured under the following conditions: normal medium (blank control), HMEC‐1 medium exosomes (C) and CHP HMEC‐1 medium exosomes (CHP). The exosome suspension was diluted in SH‐SY5Y medium at a ratio of 1:100, resulting in a concentration of approximately 35 µg exosomes/mL of medium (Cui et al., 2020). Subsequently, the cells were subjected to oxygen–glucose deprivation/reperfusion (OGD/R). OGD conditions (5% CO_2_, 1% O_2_, medium without serum and glucose; 37°C) were maintained for 6 h, followed by reperfusion (introduction of oxygen and glucose) for another 24 h (Li & Ma, 2020).

### 2.15 Cell viability assays and cell cycle detection

SH‐SY5Y cells were plated in dishes and cultured under standard conditions (5% CO_2_, 21% O_2_, 37°C). For MTS assays, SH‑SY5Y cells were seeded at a density of 2 × 10^3^ cells per well in 96‑well plates. After 24 h, the cells were incubated with exosomes for another 24 h and then exposed to OGD/R, followed by reperfusion. Viability of SY5Y cells was assessed using the MTS assay as previously described (Beyotime Biotechnology) (Tian et al., 2019). Briefly, 20 µL of MTS reagent was added to each well and incubated for 2 h at 37°C. Absorbance was measured every 30 min at 490 nm using a microplate reader. A minimum of 1 × 10^4^ cells per sample was used for flow cytometry. SY5Y cells were collected following incubation with exosomes and OGD/R treatment. Cell pellets were resuspended in 10× Annexin V Binding Buffer and stained with 5 µL fluorescein isothiocyanate (FITC) Annexin V and 2 µL propidium iodide. The percentages of cells in different phases and the percentage of apoptotic cells were analysed via FACScan flow cytometry (BD Biosciences, San Jose, CA, USA) using a ModFit 3.0 computer program (BD Biosciences). The experiments were conducted using three independent biological replicates (i.e., separate culture medium batches), and each sample was analysed in triplicate.

### 2.16 Statistical analysis

Data are presented as means ± standard deviation (SD). Statistical analyses were performed using GraphPad Prism version 8.0.1 (GraphPad Software, San Diego, CA, USA) and SPSS (version 21.0; IBM Corp., Armonk, NY, USA). For large‐scale miRNA differential expression analyses (e.g., next‐generation sequencing data), the false discovery rate was controlled using the Benjamini–Hochberg procedure. For comparisons between two groups, a two‐sided independent samples Student's *t*‐test or Mann–Whitney *U*‐tests was applied, depending on data distribution. For comparisons of more than two groups, one‐way ANOVA was applied followed by appropriate *post hoc* tests. Categorical variables were summarized as frequencies and percentages, and differences between groups were assessed using the χ^2^ or Fisher's exact test, as appropriate. *P *< 0.05 was considered to indicate a statistically significant difference.

## 3 RESULTS

### 3.1 RIPC‐induced dicrotic wave

**FIGURE 1:**
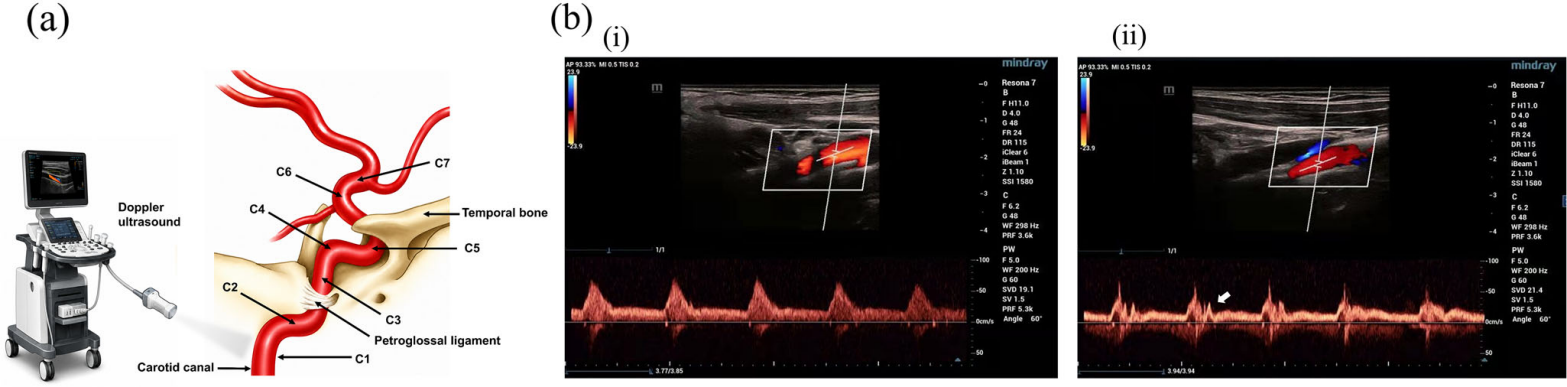
Doppler blood flow shows the waveforms obtained from the internal carotid artery of a volunteer. (a) Schematic diagram shows the location of the internal carotid artery for Doppler sonography. (b) Representative Doppler sonograms of the internal carotid artery: (i) waveform without a dicrotic wave, recorded before RIPC; (ii) waveform with a dicrotic wave (white arrow) recorded during the tense phase of the third RIPC cycle.

All participants had a 1‐h rest period prior to RIPC. During the study period, 9 of the 10 participants who underwent RIPC exhibited a dicrotic wave (Figure 1). No dicrotic waves were observed before RIPC in any participant (Table 1). During the release phase of the first cycle, a dicrotic wave was induced in 50% of the participants, and it was also induced during subsequent phases of RIPC, ranging from 20% to 50% of the treatment process (Table 1). The same individual did not consistently exhibit the dicrotic wave throughout the study. No significant differences were observed throughout the entire RIPC process in the following parameters: systolic peak, end‐diastolic velocity, TAMAX and TAMEAN, pulsatility index, resistance index, systole/diastole ratio and body flow volume.

**TABLE 1:** Percentage of participants exhibiting a dicrotic wave before and after RIPC.

Cycle		1		2		3		4		5	
Cuff status	Baseline	T	R	T	R	T	R	T	R	T	R
Detection time point (min)	0	3	8	13	18	23	28	33	38	43	48
Men (1–5) (%)	0	20	60	40	40	80	20	20	20	60	0
Women (1–5) (%)	0	60	40	20	20	20	20	20	20	20	40
Total (%)		40	50	30	30	50	20	20	20	40	20

Abbreviations: R, release (Cuff flate); T, tense (Cuff inflate).

### 3.2 RIPC increases VWP

**FIGURE 2:**
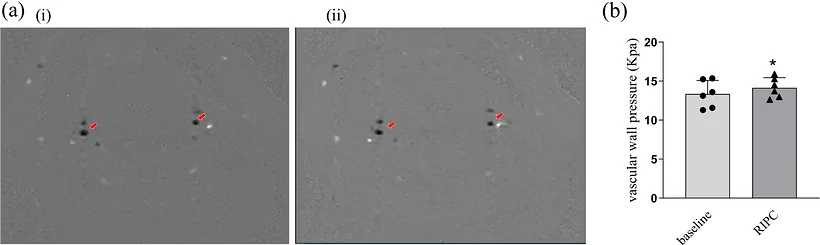
Magnetic resonance imaging (MRI) scans of the carotid segment of the internal carotid artery from a volunteer and vascular wall pressure calculated from MRI data. (a) Images from MRI scan: (i) before and (ii) after RIPC. The red arrow indicates the internal carotid artery. (b) Vascular wall pressure (calculated from MRI data). Data are expressed as means ± SD. *n* = 6 for each group. Asterisk indicates statistically significant difference compared with baseline.

A typical MRI phase image of the internal carotid artery was obtained from a volunteer before and after RIPC (Figure 2a). The mean VWP value of the participants immediately after RIPC was 14.15 ± 1.29 kPa, which was higher than the mean VWP value (13.36 ± 1.74 kPa) at baseline (*n* = 6; *P* = 0.0137; Figure 2b). These results indicate that RIPC can increase VWP.

**FIGURE A1:**
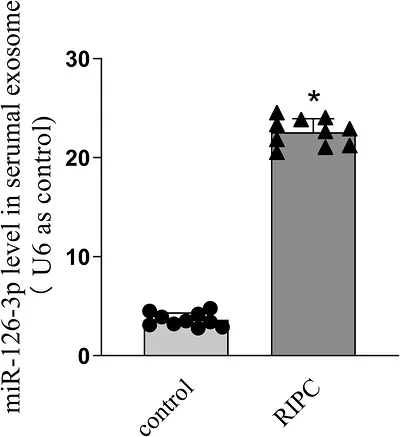
RIPC‐treatment increased miRNA‐126‐3p level in serum exosomes. Data are expressed as means ± SD. n = 10 for each group. Asterisk indicates statistically significant difference compared with control group.

Serum exosomal miR‐126 was significantly increased after RIPC (*n* = 10; *P *< 0.0001; Appendix Figure A1), which is consistent with the observations in our earlier pilot study (Cui et al., 2020; *n* = 3) and findings from our in vitro studies indicating that cyclic pressure upregulates endothelial miR‐126.

### 3.3 CHP increases miR‐126 levels and decreases miR‐126 promoter DNA methylation in HMEC‐1 cells

**FIGURE 3:**
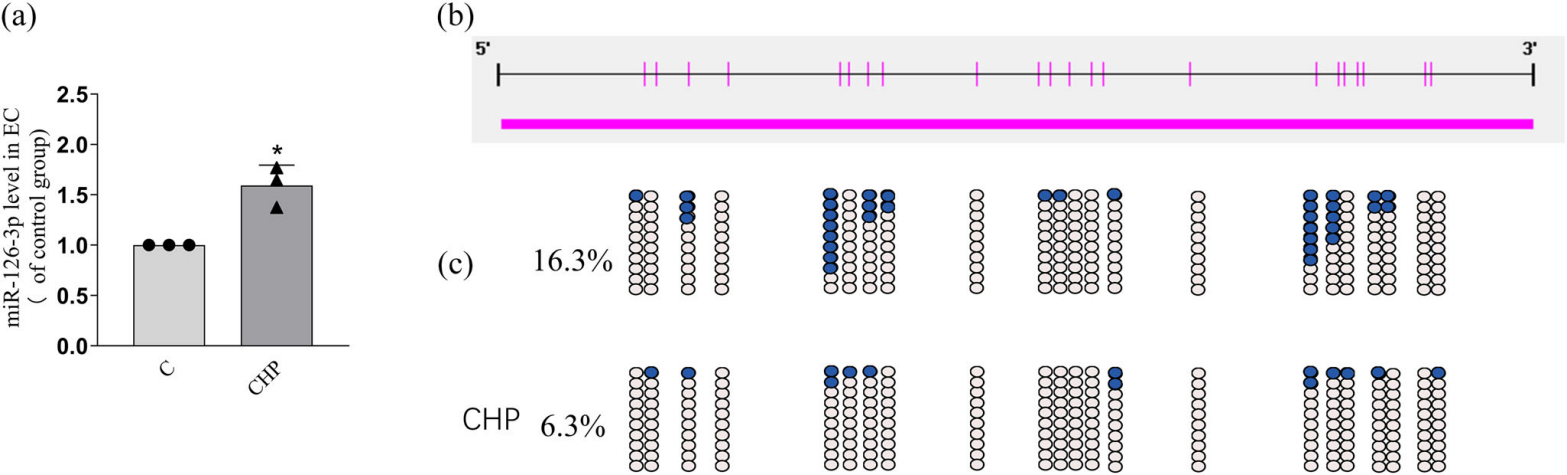
Effect of CHP on the expression and CpG island methylation of miR‐126‐3p in HMEC‐1 cells. (a) Relative expression of miR‐126‐3p. (b) The changes of CpG island methylation level of miR‐126‐3p analysed by BMDS. Ten single clones are shown for each sample. The CpG island is depicted, with each circle representing a single CpG site. Filled circles indicate methylated CpGs; open circles indicate unmethylated CpGs. Data are expressed as means ± SD. *n* = 3 for each group. The asterisk indicates statistically significant difference compared with control group. BMDS, bisulfite‐modified DNA sequencing; CHP, cyclic higher pressure; HMEC‐1, human microvascular endothelial cell‐1.

In HMEC‐1 cells treated with elevated pressure, miRNA‐126 expression was significantly higher than that in the control group (*n* = 3, *P* = 0.0070; Figure 3a). HMEC‐1 cells exposed to CHP exhibited elevated miR‐126 expression levels and fewer methylated promoters than the control group (Figure 3b).

### 3.4 CHP decreases DNMT3A and DNMT3B expression and activity in HMEC‐1 cells

**FIGURE 4:**
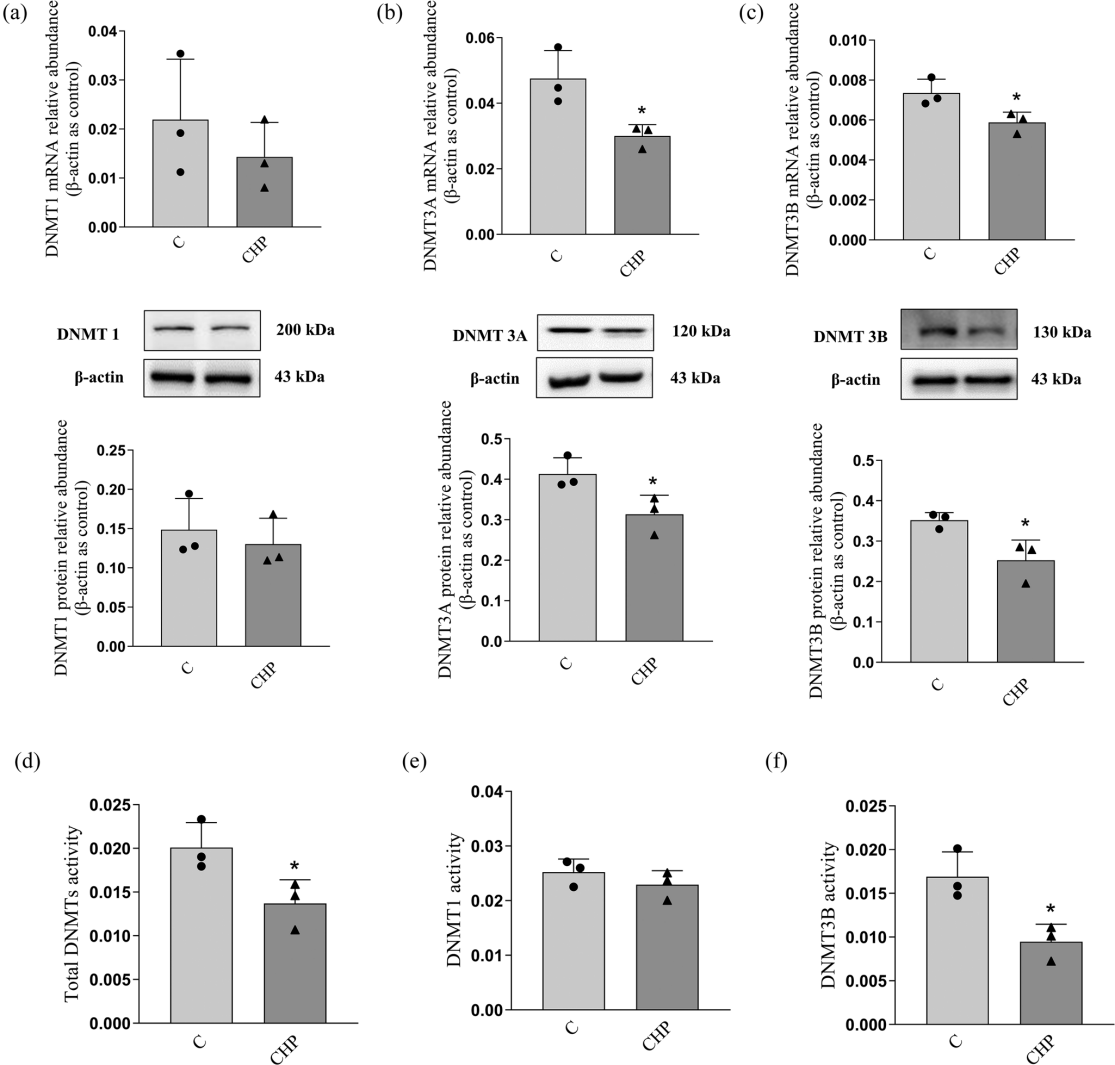
The effect of CHP on the expression and activity of DNMTs in HMEC‐1. (a–c) mRNA and protein levels measured by RT‐qPCR and western blotting showing reduction in DNMT3A and DNMT3B but not DNMT1. (d–f) Activity of total DNMTs, DNMT1 and DNMT3B measured by ELISA‐based activity assay. Data are expressed as means ± SD. *n* = 3 for each group. Asterisk indicates statistically significant difference compared with control group. CHP, cyclic higher pressure; DNMT, DNA methyltransferase; HMEC‐1, human microvascular endothelial cell‐1.

To explore the possible involvement of DNMTs in increasing miR‐126 expression in HMEC‐1 cells following CHP exposure, the expression of three major DNMTs was analysed. In HMEC‐1 cells exposed to CHP, mRNA expression of DNMT3A (*P* = 0.0309) and DNMT3B (*P* = 0.0420) decreased, whereas that of DNMT1 remained unchanged (*P* = 0.4051; Figure 4a–c). Furthermore, when comparing the CHP group with the control group, a significant downregulation of DNMT3A (*P* = 0.0486) and DNMT3 proteins was observed (*P* = 0.0331; Figure 4b, c). There were no significant differences in the DNMT1 protein (*P* = 0.5748) levels between the two groups (Figure 4a).

The enzymatic activity of DNMTs in HMEC‐1 cells was assessed using ELISA. In the CHP group, total DNMT (*P* = 0.0476) and DNMT3B (*P* = 0.0207) activity was reduced, whereas DNMT1 (*P* = 0.3236) activity remained unchanged (Figure 4d–f).

### 3.5 CHP reduces Alu and LINE‐1 methylation levels in HMEC‐1 cells

**FIGURE 5:**
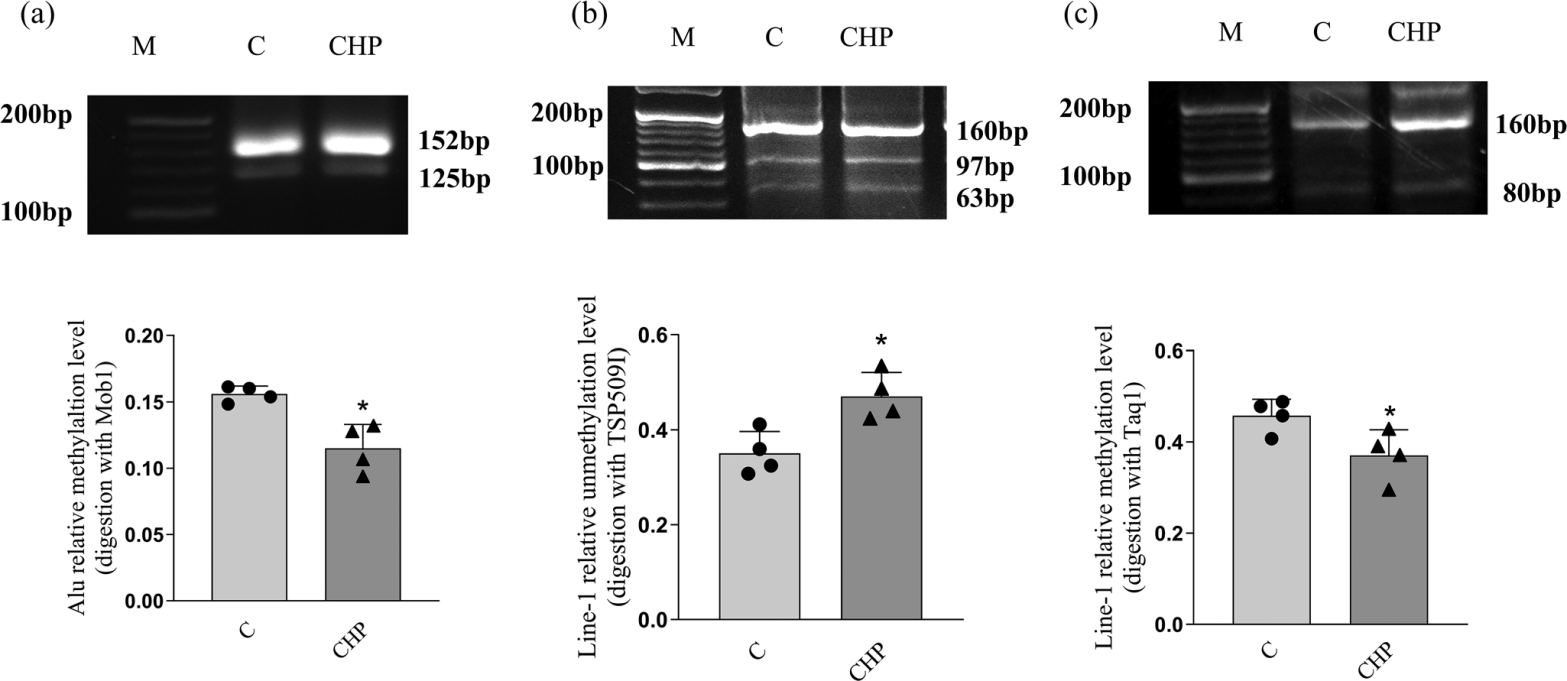
Level of genomic methylation in HMEC‐1 reduced following CHP treatment. (a) Representative images of COBRA products (Alu sequence + *Mbo*I digestion) separated by agarose gel electrophoresis, and the DNA methylation level in the Alu sequence (methylation ratio = 125 bp/[125 bp + 152 bp]). (b) Representative images of COBRA products (LINE‐1 sequence + Tsp509I digestion) separated by agarose gel electrophoresis, and the unmethylated DNA level in the LINE‐1 sequence (unmethylated ratio = [63 bp + 97 bp]/[63 bp + 97 bp + 160 bp]). (c) Representative images of COBRA products (LINE‐1 sequence + *Taq*I digestion) separated by agarose gel electrophoresis, and the DNA methylation level in the LINE‐1 sequence (methylation ratio = 80 bp/[80 bp + 160 bp]). Data are expressed as means ± SD. *n* = 4 for each group. Asterisk indicates statistically significant difference compared with the control group. CHP, cyclic higher pressure; COBRA, combined bisulfite restriction analysis; HMEC‐1, human microvascular endothelial cell‐1; LINE‐1, long interspersed nuclear element‐1.

LINE‐1 and Alu repetitive DNA elements are commonly used reliable indicators of global DNA methylation levels. The relative methylation level of the Alu sequence was 0.156 ± 0.006 in the control group and 0.115 ± 0.018 in the CHP group (*P* = 0.0050; Figure 5a). The LINE‐1 unmethylation level was 0.351 ± 0.046 in the control group and 0.471 ± 0.050 in the CHP group (*P* = 0.0123; Figure 5b). The LINE‐1 methylation level was 0.458 ± 0.036 and 0.371 ± 0.056 in the control and CHP groups, respectively (*P* = 0.0399; Figure 5c). These results indicated that CHP reduced global methylation levels.

### 3.6 Exosomes isolated from culture medium

**FIGURE 6:**
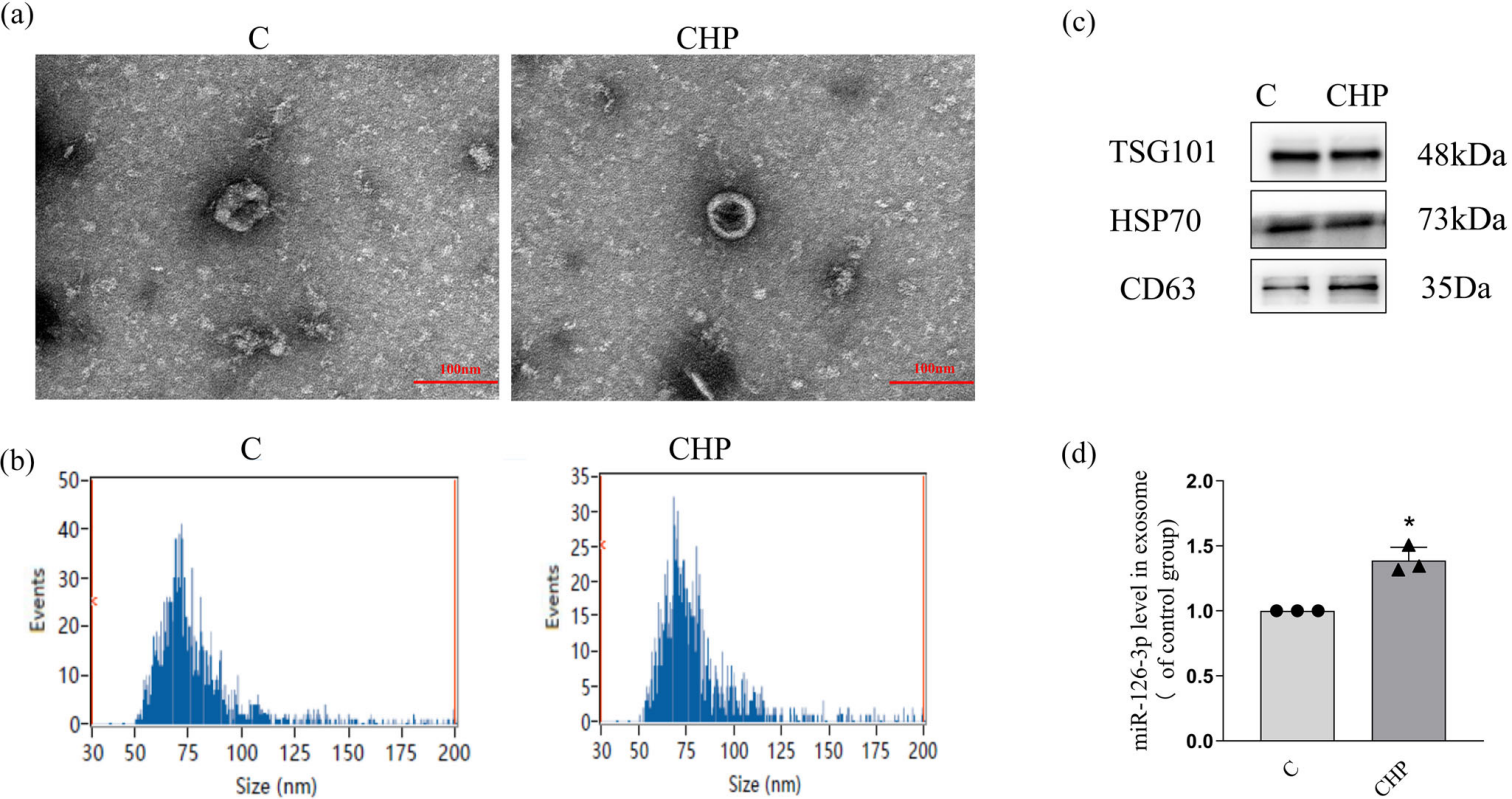
Exosome characterization and miR‐126‐3p levels in exosomes. (a) Representative transmission electron microscopy images of exosomes (scale bar: 100 nm). (b) Cumulative analysis showed the size distribution of exosomes. (c) Representative western blots showed exosomal markers HSP70, TSG101 and CD63. (d) Relative expression of miR‐126‐3p in exosomes isolated from culture medium of control and CHP‐treated HMEC‐1 cells. Data are expressed as means ± SD. *n* = 3. Asterisk indicates statistically significant difference compared with control group. CHP, cyclic higher pressure.

To confirm that the structures within the culture medium were exosomes, they were examined using electron microscopy and western blotting. The electron microscopy results showed that the isolated pellets had a spherical, ellipsoidal or tea‐tray shape, with sizes of 40–150 nm. This size range aligned with the typical dimensions of exosomes (Figures 6a, b). Western blotting was confirmed the presence of three specific exosomal protein markers – CD63, HSP701 and TSG10 – in the isolated pellets (Figure 6c). miR‐126 levels in the exosomes were measured in real time and were found to be increased in the CHP group (*P* = 0.0028; Figure 6d).

### 3.7 Differentially expressed miRNAs in exosomes and HMEC‐1 cells and functional analysis of miRNA target genes

**FIGURE 7:**
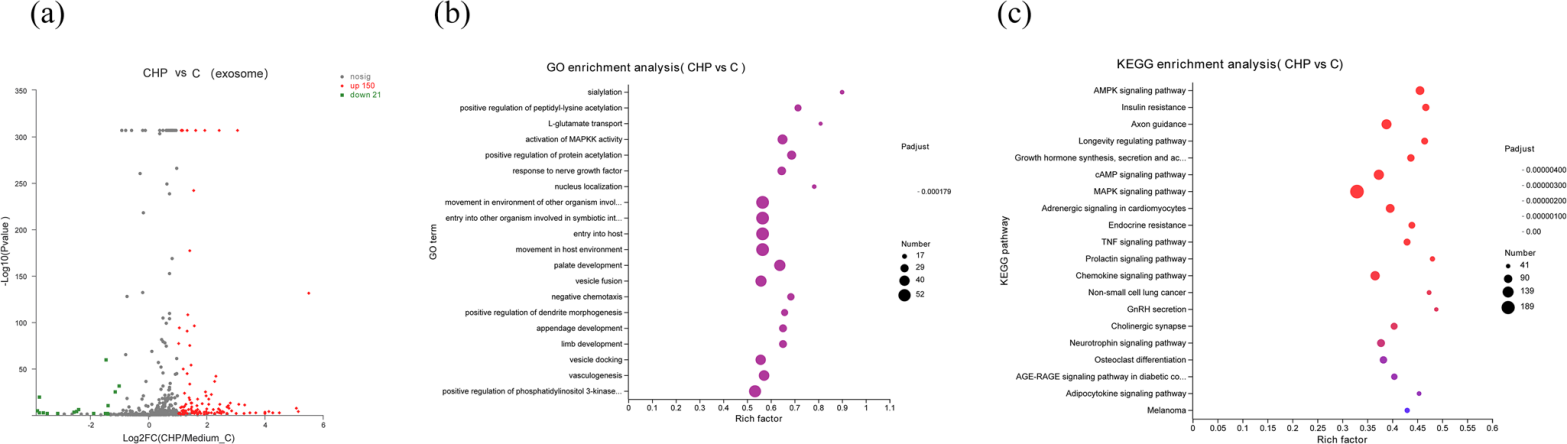
Differential expression of miRNAs in exosomes from culture medium of HMEC‐1 cells. (a) Volcano plot showing differentially expressed miRNAs. (b) GO enrichment analysis of differentially expressed miRNAs. (c) KEGG pathway enrichment analysis of differentially expressed miRNAs. GO, Gene Ontology; KEGG, Kyoto Encyclopedia of Genes and Genomes; miRNA, microRNA.

Within the exosomes, 150 miRNAs were upregulated in the CHP group, whereas 21 miRNAs were downregulated compared to those in the control group (Figure 7a). Gene Ontology (GO) and Kyoto Encyclopedia of Genes and Genomes (KEGG) analyses were performed to explore the functions of differentially expressed miRNAs (Figures 7b, c). GO analysis revealed that these miRNAs were associated with functions related to cytoplasmic vesicles, cell cycle and cell projection. KEGG analysis, on the other hand, indicated the involvement of these miRNAs in pathways such as hypoxia‐inducible factor 1 (HIF‐1) signalling, forkhead box O (FOXO) signalling and vascular smooth muscle contraction.

### 3.8 Exosomes from CHP‐treated HMEC‐1 medium exert neuroprotection against OGD/R in SH‐SY5Y cells

**FIGURE 8:**
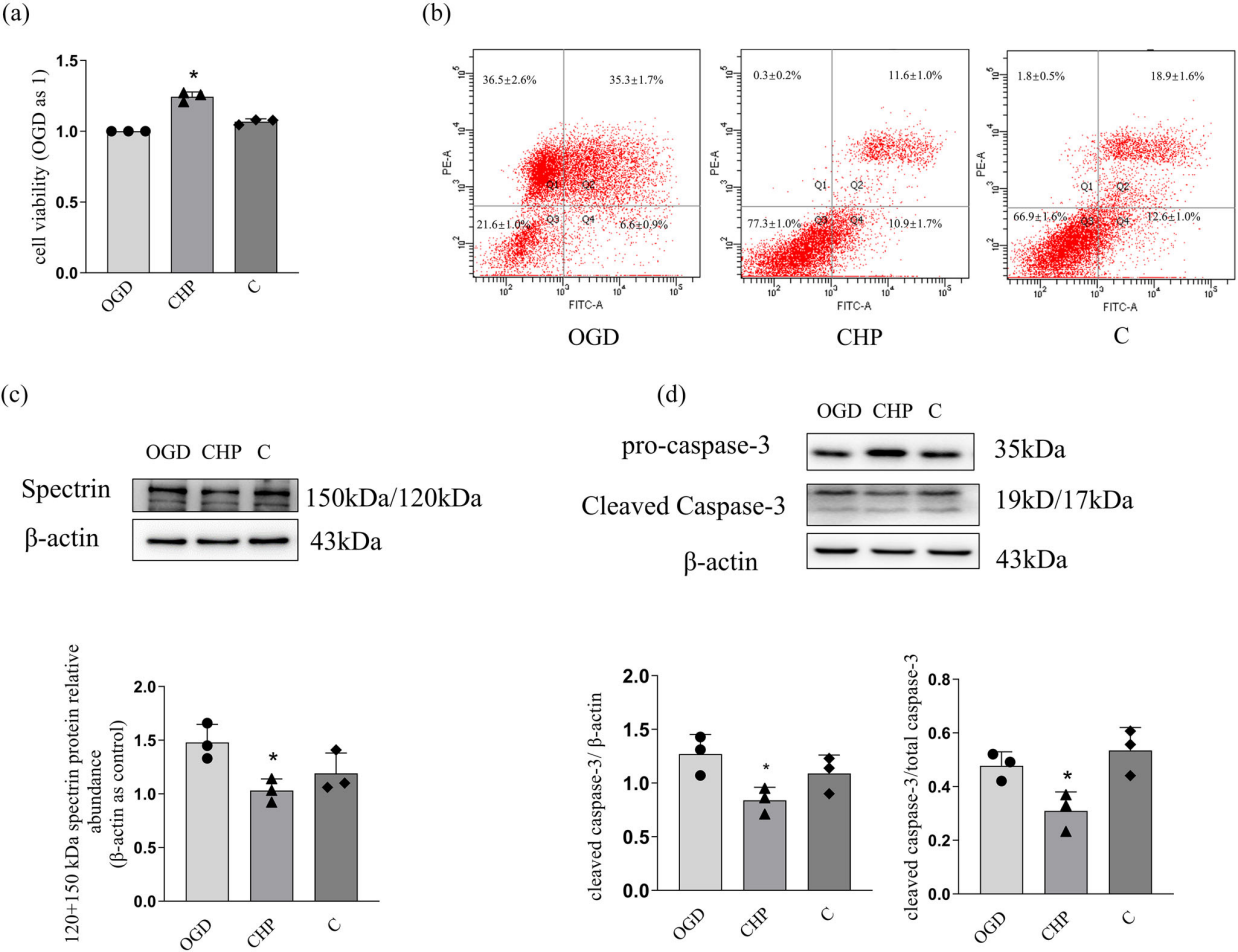
Exosomes from CHP‐treated HMEC‐1 culture medium showed protective effects in SH‐SY5Y cells under OGD/R conditions. (a) Cell viability following OGD measured by MTS assay showed significant difference after CHP treatment. (b) Flow cytometry showed cell apoptosis changed following CHP treatment. (c) Western blot analysis showed spectrin degradation among 3 groups. Upper panel: representative images of western blot for spectrin. Lower panel: statistic analysis showed spectrin level was significant lower following CHP treatment. (d) Western blot analysis showed cleaved caspase‐3 reduced following CHP treatment. Upper panel: representative images of western blot for caspase‐3. Lower panel: statistical analysis showed cleaved caspase‐3 level was significant lower following CHP treatment. Data are expressed as means ± SD. *n* = 3 for each group. Asterisk indicates statistically significant difference compared with OGD group. CHP, cyclic higher pressure; HMEC‐1, human microvascular endothelial cell‐1; OGD/R, oxygen‐glucose deprivation/reperfusion.

The neuroprotective effect of these exosomes was assessed by evaluating the viability of SH‐SY5Y cells using the MTS assay. Compared with the OGD/R (normalized to 1.00) and control (1.11 ± 0.03) groups, cell viability was significantly higher in the CHP group (1.30 ± 0.05) (*P *< 0.0001; Figure 8a).

Apoptosis rates were determined using flow cytometry. The results showed that, relative to the OGD/R group (35.3 ± 1.7%), both the CHP (11.6 ± 1.0%) and control (18.9 ± 1.6%) groups exhibited significantly reduced late apoptosis rates (*P *< 0.0001). Notably, the CHP group also demonstrated a significantly lower rate of late apoptosis than the control group (*P* = 0.0023; Figure 8b).

### 3.9 Exosomes from CHP‐treated HMEC‐1 culture medium reduce spectrin degradation and decreases cleaved caspase‐3 levels under OGD/R conditions in SH‐SY5Y cells

Spectrin cleavage produces two distinct breakdown products: one with a size of 150 kDa, indicating cell necrosis and excitotoxic neuronal death, and another with a size of 120 kDa, indicating apoptotic cell death (Wang et al., 2018). In the CHP group subjected to OGD/R, the expression of spectrin breakdown products at 150 kDa + 120 kDa decreased (*P* = 0.0358; Figure 8c).

Cleaved caspase‐3 (19/17 kDa) levels, which can serve as an indicator of apoptotic activity in cells or tissues, were decreased in the CHP group (cleaved caspase‐3/β‐actin, *P* = 0.0452; cleaved caspase‐3/total caspase‐3, *P* = 0.0118; Figure 8d).

## 4 DISCUSSION

RIPC is currently considered a promising method for ischaemic protection and may have clinical applications (Zhu et al., 2025). It has been suggested that endogenous protective molecules are released and act on target organs to promote cellular protective processes (Mollet et al., 2022). Therefore, the components of RIPC may include sensors of stress signals, stimulus transducers or effectors of tolerance (Mollet et al., 2022). RIPC upregulates serum exosomal miRNA‐126, which is released by ECs (Cui et al., 2020). Corti & Gladwin (2014) have presented compelling evidence that limb ischaemia induces metabolic vasodilation, leading to increased blood flow and shear forces on the endothelium of conductance blood vessels, thereby activating eNOS. Therefore, ECs may serve as RIPC sensors, triggering the release of endogenous protective molecules.

The pressure that triggers the release of protective factors by ECs is thought to originate primarily from arterial pressure, particularly its phasic components (Pagliaro et al., 1999, 2000). One such phasic phenomenon is the dicrotic wave. Lewis (1906) reported that a patient with a systolic blood pressure of 180 mmHg may have a palpable dicrotic wave pulse. Chai et al. (2017). provided clinical data and numerical simulations supporting the idea that reflected pressure waves can contribute to the dicrotic wave. The dicrotic wave is a well‐known feature in the descriptive analysis of arterial waveforms and has been frequently used as a proxy for end‐systolic left ventricular pressure (Politi et al., 2016). Its characteristics also depend on the duration of ejection as well as body size and shape (Hussain & Ahmed, 1997). Given these associations between the dicrotic wave and arterial pressure, an accurate assessment of central haemodynamics, including central blood pressure waveforms, is very important in understanding the dicrotic wave and its physiological implications (Gao et al., 2016). The VWP was calculated from the MRI data, and was found to be increased in the present study. These results were similar to those of Hebert et al. (1995), who reported that dicrotic wave pressure exceeded the mean pressure when using a high‐fidelity transducer. In our study, the mean VWP calculated using MRI data was found to be increased after RIPC. Thus, the dicrotic wave corresponds to a transient pressure increase of approximately 5–10 mmHg (Hebert et al., 1995; Pal et al., 2024). In contrast, our in vitro CHP model applied a sustained pressure elevation (100 mmHg above atmospheric pressure) for 5 min per cycle. We acknowledge these substantial differences in both the magnitude and temporal dynamics. Importantly, our reductionist model does not attempt to replicate the physiological pulse waveform, but rather to isolate the effects of cyclic mechanical pressure from other RIPC‐associated factors such as hypoxia and shear stress. Although the magnitude of the applied pressure exceeds that observed in vivo, repeated cycles of RIPC‐induced mechanical stimulation may induce cumulative epigenetic modifications (for example, DNMT downregulation and miR‐126 demethylation) which contribute to neuroprotection. Nonetheless, the physiological effects of RIPC on ECs are likely complex, and future studies must integrate pressure changes with hypoxia/reoxygenation and flow‐based shear stress to better approximate the in vivo situation.

Biomechanical forces resulting from blood flow, such as pressure, stretch and shear stress, can influence EC function by regulating gene expression (Tsuboi et al., 1995; Zhou et al., 2012). Zhou et al. (2013) reported that laminar shear stress (LSS) did not affect miR‐126 expression in ECs. Conversely, disturbed blood flow leads to the upregulation of miR‐126‐3p both in vitro and in vivo (Liu et al., 2019). Previous studies have shown that circulating miR‐126 levels are significantly lower in patients with hypertension than in normotensive individuals (Kontaraki et al., 2014; Marketou et al., 2019; Suzuki et al., 2021). However, there are contrasting results: Liu et al. (2018) discovered that miR‐126 expression was higher in patients with hypertension than in healthy individuals. Although the reasons for this discrepancy require further investigation, it is evident that blood pressure can affect miR‐126 expression in ECs. Therefore, in the present study, we focused on the effects of pressure on ECs and observed that CHP enhanced miR‐126 expression in cultured ECs.

miR‐126 is involved in adaptive alterations in vessel wall remodelling induced by arterial pressure (Nazari‐Jahantigh et al., 2012). The inhibition of miR‐126‐3p exacerbates cell death induced by OGD/R and reduces cell viability (Xiao et al., 2020). It has been shown that SH‐SY5Y cells incubated with culture medium from HMEC‐1 cells overexpressing miR‐126‐3p exhibit greater OGD/R tolerance (Zhang et al., 2024). In the present study, exosomal miR‐126‐3p in the medium in which CHP‐treated ECs were cultured increased the resilience of SH‐SY5Y cells against OGD/R. HTS results indicated that CHP upregulated exosome miRNA‐targeting pathways, such as FOXO. Increased miR‐122 expression protects against neuronal cell death following ischaemic stroke. This protective effect occurs through the HSP70‐dependent nuclear factor‐κB pathway, which is mediated by FOXO3 (Guo et al., 2018). FOXO3 silencing markedly inhibits neuronal cell apoptosis under OGD conditions (Deng et al., 2020). It has also been reported that endothelial miR‐126 suppresses FOXO3 expression (Zhou et al., 2013). Therefore, miR‐126 overexpression may increase OGD/R tolerance through the FOXO3 pathway.

miR‐126 expression may depend on DNA methylation levels. Polyunsaturated fatty acids increase miR‐126 expression by demethylating the miR‐126 promoter (Moradi Sarabi et al., 2018). In addition, Potus et al. (2015) showed that right ventricular hypertrophy is associated with highly methylated promoters and decreased expression of miR‐126. However, hypoxia did not affect miR‐126 expression. In our previous study, RIPC treatment increased exosomal miR‐126 levels in healthy participants (Cui et al., 2020). In the present study, five cycles of high‐pressure treatment induced miR‐126 expression in vitro. Furthermore, repeated high‐pressure treatment reduced DNA methylation levels of the miR‐126 promoter. These findings indicate that RIPC‐induced upregulation of miR‐126 may be partly owing to changes in force rather than solely because of hypoxia in EC.

DNMTs regulate gene promoter methylation levels. Decreased methylation of miR‐126 has been linked to increased expression of DNMT3A and DNMT3B (Potus et al., 2015). In vivo and in vitro results of a study by Zhou et al. (2014) demonstrated the differential regulation of DNMT1 and DNA hypermethylation by different haemodynamics in ECs. Oscillatory disturbed flow (DF) in vitro induced an increase in DNMT3A protein levels and did not affect the expression of DNMT1 and DNMT3B in ECs (Jiang et al., 2014). Considering the above findings, we propose that biomechanical forces, such as blood vessel wall pressure, may induce changes in DNMT expression. In the present study, subjecting ECs to cyclic pressure resulted in a downregulation of DNMT3A and DNMT3B, which may be responsible for decreased miR‐126 methylation levels. However, direct causal testing of this correlation model, particularly with regard to the essential role of miR‐126, is required in future studies.

The present study has several limitations. First, the relatively small sample size of healthy young volunteers may limit the generalizability of our findings, particularly to patients with cerebrovascular diseases, who are the primary targets for RIPC therapy. Future studies with larger cohorts including such patients are warranted. Second, the use of HMEC‐1, which is a microvascular endothelial line, may not fully recapitulate the responses of macrovascular ECs or their complex in vivo environment. Additionally, the SH‐SY5Y neuroblastoma cells used in this study do not adequately reflect primary neuronal physiology. Therefore, validation in primary human macrovascular ECs (HUVECs) and in more physiologically relevant neuronal (e.g., primary neurons or iPSC‐derived neurons) or animal models is critically important. Finally, although we observed an inverse correlation between DNMT3A/B expression and miR‐126 levels, we were unable to establish a causal relationship. Direct functional evidence from loss‐ or gain‐of‐function experiments (miR‐126 inhibition/overexpression and DNMT3A/B siRNA/overexpression) will be required to prove necessity or sufficiency in future work.

In conclusion, the present findings demonstrated that RIPC transiently increases VWP. Repeated transient elevation of blood pressure in ECs results in the downregulation of DNMT expression and activity, leading to an upregulation of miRNA‐126. In turn, exosomes from CHP‐exposed ECs, which have higher miRNA‐126 levels, exhibit a protective effect against OGD/R injury in cultured nervous cells.

## AUTHOR CONTRIBUTIONS

Methodology: Xiaojie Wang, Qi Liu, Hongwei Zhu, Lei Yan and Yifan Li. Investigation: Zhijun Zhao and Chunyang Zhang; writing – review and editing: Wei Li and Kerui Gong; supervision: Gang Fu and Xunming Ji; writing‐original draft, funding acquisition: Guo Shao. All authors have read and approved the final version of this manuscript and agree to be accountable for all aspects of the work in ensuring that questions related to the accuracy or integrity of any part of the work are appropriately investigated and resolved. All persons designated as authors qualify for authorship, and all those who qualify for authorship are listed.

## CONFLICT OF INTEREST

None declared.

## GENERATIVE AI STATEMENT

The authors declared that no generative AI tools were used in any aspect of this work.

## Data Availability

The microRNA sequencing datasets generated and analysed during the current study are available in the NCBI Sequence Read Archive (SRA) repository, accession number PRJNA1302644(http://www.ncbi.nlm.nih.gov/bioproject/1302644.). Other data generated in the present study are included in the figures and/or tables of this article.

## 1

**TABLE A1:** Primers for RT‐PCR.

Gene	Forward primer (5′ → 3′)	Reverse primer (5′ → 3′)
miRNA‐126‐3P	TCGTCTGCCGTACCGTGAGTAAT	CACTTCCTCAGCTCTTGTTGGTAT
U6‐Hu	CGCTTCGGCAGCACATATACTA	CGCTTCACGAATTTGCGTGTCA
DNMT1	AACCTTCACCTAGCCCCAG	CTCATCCGATTTGGCTCTTTCA
DNMT3A	GACAAGAATGCCACCAAAGC	CGTCTCCGAACCACATGAC
DNMT3B	AGGGAAGACTCGATCCTCGTC	GTGTGTAGCTTAGCAGACTGG
β‐Actin	CATGTACGTTGCTATCCAGGC	CTCCTTAATGTCACGCACGAT

*Note*: The reverse primer sequence for miR‐126‐3p is part of the stem‐loop RT system provided in the Hairpin‐it™ miRNA RT‐PCR kit (GenePharma). It was designed to anneal into a stem‐loop structure. This primer served as a universal reverse primer in qPCR reactions and was used together with miRNA‐specific forward primers.

**TABLE A2:** Primer for mir‐126 bisulfite sequencing PCR.

Gene	Forward primer (5′ → 3′)	Reverse primer (5′ → 3′)
miR‐126‐O	**T**AGGGAGAGGTGAGAGGAAAG	CCR**AA**CCTCCCR**A**ACCCCTCA
miR‐126‐I	AAAGG**T**TGGGGA**TTT**T**T**A**T**T	TCACCA**AAA**T**A**CTTTT**AA**CCTCT**A**A

*Note*: Bold bases in the forward primers indicate C→T conversions; bold bases in the reverse primers indicate G→A conversions (the bisulfite reaction). ‘R’ denotes G or A, corresponding to the original methylated or unmethylated cytosine, respectively.

**TABLE A3:** Primers for Alu and line‐1.

Gene	Forward primer (5′ → 3′)	Reverse primer (5′ → 3′)
LINE‐1	CCG**T**AAGGGGT**T**AGGGAGTT**TTT**	RTA**AA**ACCCTCCRA**A**CCA**AA**T**A**T**AAA**
Alu	GATCT**T**T**T**TA**T**TAAAAATA**T**AAAAATTAG**T**	GATCCCA**AA**CT**AA**A**A**T**A**CA**A**T**AA**

*Note*: Bold bases in the forward primers indicate C→T conversions; bold bases in the reverse primers indicate G→A conversions (the bisulfite reaction). ‘R’ denotes G or A, corresponding to the original methylated or unmethylated cytosine, respectively.

## References

[eph70462-bib-0001] Andersen, M. , Trapani, D. , Ravn, J. , Sorensen, J. B. , Andersen, C. B. , Grauslund, M. , & Santoni‐Rugiu, E. (2015). Methylation‐associated Silencing of microRNA‐126 and its host gene EGFL7 in malignant pleural mesothelioma. Anticancer Research, 35(11), 6223–6229. 26504055

[eph70462-bib-0002] Anttila, T. , Herajärvi, J. , Laaksonen, H. , Mustonen, C. , Honkanen, H.‐P. , Y Dimova, E. , Piuhola, J. , Koivunen, P. , Juvonen, T. , & Anttila, V. (2023). Remote ischemic preconditioning and hypoxia‐induced biomarkers in acute myocardial infarction: Study on a porcine model. Scandinavian Cardiovascular Journal, 57(1), 2251730. 37641930 10.1080/14017431.2023.2251730

[eph70462-bib-0003] Anwar, M. A. , Shalhoub, J. , Lim, C. S. , Gohel, M. S. , & Davies, A. H. (2012). The effect of pressure‐induced mechanical stretch on vascular wall differential gene expression. Journal of Vascular Research, 49(6), 463–478. 22796658 10.1159/000339151

[eph70462-bib-0004] Bannell, D. J. , Montrezol, F. T. , Maxwell, J. D. , Somani, Y. B. , Low, D. A. , Thijssen, D. H. J. , & Jones, H. (2023). Impact of handgrip exercise and ischemic preconditioning on local and remote protection against endothelial reperfusion injury in young men. American Journal of Physiology‐Regulatory, Integrative and Comparative Physiology, 324(3), R329–R335. 36572551 10.1152/ajpregu.00061.2022

[eph70462-bib-0005] Chai, R. , Li, S.‐M. , Xu, L.‐S. , Yao, Y. , & Hao, L.‐L. (2017). Regression analysis and transfer function in estimating the parameters of central pulse waves from brachial pulse wave. Annual International Conference of the IEEE Engineering in Medicine and Biology Society, 2017, 1708–1711. 29060215 10.1109/EMBC.2017.8037171

[eph70462-bib-0006] Corti, P. , & Gladwin, M. T. (2014). Is nitrite the circulating endocrine effector of remote ischemic preconditioning? Circulation Research, 114(10), 1554–1557. 24812347 10.1161/CIRCRESAHA.114.303960 PMC4073608

[eph70462-bib-0007] Cui, J. , Liu, N. A. , Chang, Z. , Gao, Y. , Bao, M. , Xie, Y. , Xu, W. , Liu, X. , Jiang, S. , Liu, Y. , Shi, R. , Xie, W. , Jia, X. , Shi, J. , Ren, C. , Gong, K. , Zhang, C. , Bade, R. , Shao, G. , & Ji, X. (2020). Exosomal MicroRNA‐126 from RIPC Serum Is Involved in Hypoxia Tolerance in SH‐SY5Y Cells by Downregulating DNMT3B. Molecular Therapy Nucleic Acids, 20, 649–660. 32380415 10.1016/j.omtn.2020.04.008 PMC7210387

[eph70462-bib-0008] Davidson, S. M. , Riquelme, J. A. , Zheng, Y. , Vicencio, J. M. , Lavandero, S. , & Yellon, D. M. (2018). Endothelial cells release cardioprotective exosomes that may contribute to ischaemic preconditioning. Scientific Reports, 8(1), 15885. 30367147 10.1038/s41598-018-34357-z PMC6203728

[eph70462-bib-0009] Deng, Y. , Ma, G. , Gao, F. , Sun, X. , Liu, L. , Mo, D. , Ma, N. , Song, L. , Huo, X. , He, H. , & Miao, Z. (2020). SOX9 knockdown‐mediated FOXO3 downregulation confers neuroprotection against ischemic brain injury. Frontiers in Cell and Developmental Biology, 8, 555175. 33791290 10.3389/fcell.2020.555175 PMC8006459

[eph70462-bib-0010] England, T. J. , Hedstrom, A. , O'Sullivan, S. , Donnelly, R. , Barrett, D. A. , Sarmad, S. , Sprigg, N. , & Bath, P. M. (2017). RECAST (Remote Ischemic Conditioning After Stroke Trial): A pilot randomized placebo‐controlled phase II trial in acute ischemic stroke. Stroke, 48(5), 1412–1415. 28265014 10.1161/STROKEAHA.116.016429

[eph70462-bib-0011] Gao, M. , Rose, W. C. , Fetics, B. , Kass, D. A. , Chen, C. H. , & Mukkamala, R. (2016). A simple adaptive transfer function for deriving the central blood pressure waveform from a radial blood pressure waveform. Scientific Reports, 6(1), 33230. 27624389 10.1038/srep33230 PMC5021949

[eph70462-bib-0012] Guirro, E. C. O. , Leite, G. , Dibai‐Filho, A. V. , Borges, N. C. S. , & Guirro, R. R. J. (2017). Intra‐ and inter‐rater reliability of peripheral arterial blood flow velocity by means of Doppler ultrasound. Journal of Manipulative and Physiological Therapeutics, 40(4), 236–240. 28390709 10.1016/j.jmpt.2017.02.007

[eph70462-bib-0013] Guo, D. , Ma, J. , Li, T. , & Yan, L. (2018). Up‐regulation of miR‐122 protects against neuronal cell death in ischemic stroke through the heat shock protein 70‐dependent NF‐kappaB pathway by targeting FOXO3. Experimental Cell Research, 369(1), 34–42. 29715465 10.1016/j.yexcr.2018.04.027

[eph70462-bib-0014] Han, R. , Yang, X. , Ji, X. , & Zhou, B. (2024). Remote ischemic preconditioning prevents high‐altitude cerebral edema by enhancing glucose metabolic reprogramming. CNS Neuroscience & Therapeutics, 30(9), e70026. 39223758 10.1111/cns.70026 PMC11369019

[eph70462-bib-0015] He´bert, J.‐L. , Lecarpentier, Y. , Zamani, K. , Coirault, C. , Daccache, G. , Chemla, D. , Wuilliez, N. , & Larsonneur, L. (1995). Relation between aortic dicrotic notch pressure and mean aortic pressure in adults. American Journal of Cardiology, 76(4), 301–306. 7618629 10.1016/s0002-9149(99)80086-1

[eph70462-bib-0016] Hummitzsch, L. , Zitta, K. , Bein, B. , Steinfath, M. , & Albrecht, M. (2014). Culture media from hypoxia conditioned endothelial cells protect human intestinal cells from hypoxia/reoxygenation injury. Experimental Cell Research, 322(1), 62–70. 24394542 10.1016/j.yexcr.2013.12.022

[eph70462-bib-0017] Hussain, S. , & Ahmed, M. Y. (1997). Dicrotic wave and anthropometry. Indian Journal of Physiology and Pharmacology, 41, 187–188. 9142570

[eph70462-bib-0018] Jaffe, E. A. (1985). Physiologic functions of normal endothelial cells. Annals of the New York Academy of Sciences, 454, 279–291. 3907467 10.1111/j.1749-6632.1985.tb11868.x

[eph70462-bib-0019] Jia, P. , Ji, Q. , Zou, Z. , Zeng, Q. , Ren, T. , Chen, W. , Yan, Z. , Shen, D. , Li, Y. , Peng, F. , Su, Y. , Xu, J. , Shen, B. , Luo, Z. , Wang, C. , & Ding, X. (2024). Effect of delayed remote ischemic preconditioning on acute kidney injury and outcomes in patients undergoing cardiac surgery: A randomized Clinical Trial. Circulation, 150(17), 1366–1376. 39319450 10.1161/CIRCULATIONAHA.124.071408 PMC11495536

[eph70462-bib-0020] Jiang, Y. I.‐Z. , Jiménez, J. M. , Ou, K. , McCormick, M. E. , Zhang, L.‐D. I. , & Davies, P. F. (2014). Hemodynamic disturbed flow induces differential DNA methylation of endothelial Kruppel‐Like Factor 4 promoter in vitro and in vivo. Circulation Research, 115(1), 32–43. 24755985 10.1161/CIRCRESAHA.115.303883 PMC4065854

[eph70462-bib-0021] Katoh, K. (2023). Effects of mechanical stress on endothelial cells in situ and in vitro. International Journal of Molecular Sciences, 24(22), 16518. 38003708 10.3390/ijms242216518 PMC10671803

[eph70462-bib-0022] Keevil, H. , Phillips, B. E. , & England, T. J. (2024). Remote ischemic conditioning for stroke: A critical systematic review. International Journal of Stroke, 19(3), 271–279. 37466245 10.1177/17474930231191082 PMC10903142

[eph70462-bib-0023] Kontaraki, J. E. , Marketou, M. E. , Zacharis, E. A. , Parthenakis, F. I. , & Vardas, P. E. (2014). MicroRNA‐9 and microRNA‐126 expression levels in patients with essential hypertension: Potential markers of target‐organ damage. Journal of the American Society of Hypertension, 8(6), 368–375. 24794206 10.1016/j.jash.2014.03.324

[eph70462-bib-0024] Lewis, T. (1906). The factors influencing the prominence of the dicrotic wave. Journal of Physiology, 34(6), 414–429. 16992829 10.1113/jphysiol.1906.sp001165 PMC1465787

[eph70462-bib-0025] Li, J. , & Ma, L. (2020). MiR‐142‐3p attenuates oxygen glucose deprivation/reoxygenation‐induced injury by targeting FBXO3 in human neuroblastoma SH‐SY5Y Cells. World Neurosurgery, 136, e149–e157. 31863884 10.1016/j.wneu.2019.12.064

[eph70462-bib-0026] Li, M. , Chiou, K. R. , Bugayenko, A. , Irani, K. , & Kass, D. A. (2005). Reduced wall compliance suppresses Akt‐dependent apoptosis protection stimulated by pulse perfusion. Circulation Research, 97(6), 587–595. 16100043 10.1161/01.RES.0000181432.73920.b1

[eph70462-bib-0027] Liu, J. , Liu, J. , Shi, L. , Zhang, F. , Yu, L. , Yang, X. , & Cai, J. (2018). Preliminary study of microRNA‐126 as a novel therapeutic target for primary hypertension. International Journal of Molecular Medicine, 41(4), 1835–1844. 29393351 10.3892/ijmm.2018.3420 PMC5810200

[eph70462-bib-0028] Liu, M. , Li, L. , Zhu, J. , He, C. , Xu, Q. , Sun, A. , Kong, W. , Li, W. , & Zhang, X. (2019). Rapamycin attenuates a murine model of thoracic aortic aneurysm by downregulating the miR‐126‐3p mediated activation of MAPK/ERK signalling pathway. Biochemical and Biophysical Research Communications, 512(3), 498–504. 30904162 10.1016/j.bbrc.2019.03.083

[eph70462-bib-0029] Liu, Q. Y. , Cui, Y. , Li, W. , Qiu, J. , Nguyen, T. N. , & Chen, H. S. (2024). Effect of remote ischemic preconditioning on cerebral circulation time in severe carotid artery stenosis: Results from the RIC‐CCT trial. Cell Reports Medicine, 5(11), 101796. 39471820 10.1016/j.xcrm.2024.101796 PMC11604480

[eph70462-bib-0030] Love, M. I. , Huber, W. , & Anders, S. (2014). Moderated estimation of fold change and dispersion for RNA‐seq data with DESeq2. Genome Biology, 15(12), 550. 25516281 10.1186/s13059-014-0550-8 PMC4302049

[eph70462-bib-0031] Marketou, M. , Kontaraki, J. , Papadakis, J. , Kochiadakis, G. , Vrentzos, G. , Maragkoudakis, S. , Fragkiadakis, K. , Katsouli, E. , Plataki, M. , Patrianakos, A. , Chlouverakis, G. , Papanikolaou, K. , Vardas, P. , & Parthenakis, F. (2019). Platelet microRNAs in hypertensive patients with and without cardiovascular disease. Journal of Human Hypertension, 33(2), 149–156. 30375479 10.1038/s41371-018-0123-5

[eph70462-bib-0032] Maxwell, J. D. , Carter, H. H. , Hellsten, Y. , Miller, G. D. , Sprung, V. S. , Cuthbertson, D. J. , Thijssen, D. H. J. , & Jones, H. (2019). Seven‐day remote ischaemic preconditioning improves endothelial function in patients with type 2 diabetes mellitus: A randomised pilot study. European Journal of Endocrinology, 181(6), 659–669. 31614332 10.1530/EJE-19-0378

[eph70462-bib-0033] Mollet, I. , Martins, C. , Ângelo‐Dias, M. , Carvalho, A. S. , Aloria, K. , Matthiesen, R. , Viana‐Baptista, M. , Borrego, L. M. , & Vieira, H. L. A. (2022). Pilot study in human healthy volunteers on the mechanisms underlying remote ischemic conditioning (RIC)—Targeting circulating immune cells and immune‐related proteins. Journal of Neuroimmunology, 367, 577847. 35398724 10.1016/j.jneuroim.2022.577847

[eph70462-bib-0034] Mollet, I. G. , Viana‐Soares, R. , Cardoso‐Pires, C. , Soares, N. L. , Marto, J. P. , Mendonça, M. , Queiroga, C. S. F. , Carvalho, A. S. , Sequeira, C. O. , Teixeira‐Santos, L. , Fernandes, T. P. , Aloria, K. , Pereira, S. A. , Matthiesen, R. , Viana‐Baptista, M. , & Vieira, H. L. A. (2024). Identification of human circulating factors following remote ischemic conditioning (RIC): Potential impact on stroke. Free Radical Biology & Medicine, 224, 23–38. 39151835 10.1016/j.freeradbiomed.2024.08.017

[eph70462-bib-0035] Moradi Sarabi, M. , Zahedi, S. A. , Pajouhi, N. , Khosravi, P. , Bagheri, S. , Ahmadvand, H. , & Shahryarhesami, S. (2018). The effects of dietary polyunsaturated fatty acids on miR‐126 promoter DNA methylation status and VEGF protein expression in the colorectal cancer cells. Genes Nutrition, 13(1), 32. 30598703 10.1186/s12263-018-0623-5 PMC6299631

[eph70462-bib-0036] Nazari‐Jahantigh, M. , Wei, Y. , & Schober, A. (2012). The role of microRNAs in arterial remodelling. Thrombosis and Haemostasis, 107(04), 611–618. 22371089 10.1160/TH11-12-0826

[eph70462-bib-0037] Neth, P. , Nazari‐Jahantigh, M. , Schober, A. , & Weber, C. (2013). MicroRNAs in flow‐dependent vascular remodelling. Cardiovascular Research, 99(2), 294–303. 23612583 10.1093/cvr/cvt096

[eph70462-bib-0038] Pagliaro, P. (2000). Reversal of glibenclamide‐induced coronary vasoconstriction by enhanced perfusion pulsatility: Possible role for nitric oxide. Cardiovascular Research, 45(4), 1001–1009. 10728426 10.1016/s0008-6363(99)00414-9

[eph70462-bib-0039] Pagliaro, P. , Senzaki, H. , Paolocci, N. , Isoda, T. , Sunagawa, G. , Recchia, F. A. , & Kass, D. A. (1999). Specificity of synergistic coronary flow enhancement by adenosine and pulsatile perfusion in the dog. Journal of Physiology, 520(Pt 1), 271–280. 10517818 10.1111/j.1469-7793.1999.00271.x PMC2269556

[eph70462-bib-0040] Pal, R. , Rudas, A. , Kim, S. , Chiang, J. N. , Barney, A. , & Cannesson, M. (2024). An algorithm to detect dicrotic notch in arterial blood pressure and photoplethysmography waveforms using the iterative envelope mean method. Computer Methods and Programs in Biomedicine, 254, 108283. 38901273 10.1016/j.cmpb.2024.108283 PMC11323035

[eph70462-bib-0041] Paolocci, N. , Pagliaro, P. , Isoda, T. , Saavedra, F. W. , & Kass, D. A. (2001). Role of calcium‐sensitive K(+) channels and nitric oxide in in vivo coronary vasodilation from enhanced perfusion pulsatility. Circulation, 103(1), 119–124. 11136696 10.1161/01.cir.103.1.119

[eph70462-bib-0042] Peng, X. , Haldar, S. , Deshpande, S. , Irani, K. , & Kass, D. A. (2003). Wall stiffness suppresses Akt/eNOS and cytoprotection in pulse‐perfused endothelium. Hypertension, 41(2), 378–381. 12574111 10.1161/01.hyp.0000049624.99844.3d

[eph70462-bib-0043] Peng, X. , Recchia, F. A. , Byrne, B. J. , Wittstein, I. S. , Ziegelstein, R. C. , & Kass, D. A. (2000). In vitro system to study realistic pulsatile flow and stretch signaling in cultured vascular cells. American Journal of Physiology. Cell Physiology, 279(3), C797–C805. 10942730 10.1152/ajpcell.2000.279.3.C797

[eph70462-bib-0044] Politi, M. T. , Ghigo, A. , Fernández, J. M. , Khelifa, I. , Gaudric, J. , Fullana, J. M. , & Lagrée, P.‐Y. (2016). The dicrotic notch analyzed by a numerical model. Computers in Biology and Medicine, 72, 54–64. 27016670 10.1016/j.compbiomed.2016.03.005

[eph70462-bib-0045] Potus, F. , Ruffenach, G. , Dahou, A. , Thebault, C. , Breuils‐Bonnet, S. , Tremblay, È. , Nadeau, V. , Paradis, R. , Graydon, C. , Wong, R. , Johnson, I. , Paulin, R. , Lajoie, A. C. , Perron, J. , Charbonneau, E. , Joubert, P. , Pibarot, P. , Michelakis, E. D. , Provencher, S. , & Bonnet, S. (2015). Downregulation of MicroRNA‐126 contributes to the failing right ventricle in pulmonary arterial hypertension. Circulation, 132(10), 932–943. 26162916 10.1161/CIRCULATIONAHA.115.016382

[eph70462-bib-0046] Randhawa, P. K. , Bali, A. , & Jaggi, A. S. (2015). RIPC for multiorgan salvage in clinical settings: Evolution of concept, evidences and mechanisms. European Journal of Pharmacology, 746, 317–332. 25176179 10.1016/j.ejphar.2014.08.016

[eph70462-bib-0047] Reiss, I. , Kuntz, S. , Schmidt, R. , Kunz, C. , Gortner, L. , & Rudloff, S. (2004). Effect of pulmonary surfactant on TNF‐alpha‐activated endothelial cells and neutrophil adhesion in vitro. Immunobiology, 209(3), 235–244. 15518335 10.1016/j.imbio.2004.03.006

[eph70462-bib-0048] Seeger, J. P. H. , Lenting, C. J. , Schreuder, T. H. A. , Landman, T. R. J. , Timothy Cable, N. , Hopman, M. T. E. , & Thijssen, D. H. J. (2015). Interval exercise, but not endurance exercise, prevents endothelial ischemia‐reperfusion injury in healthy subjects. American Journal of Physiology. Heart and Circulatory Physiology, 308(4), H351–H357. 25416193 10.1152/ajpheart.00647.2014

[eph70462-bib-0049] Shao, Y. , Zhang, S. , Zhang, P. , Chen, J. , & Shao, G. (2025). MicroRNA‐126 as an endogenous molecule for hypoxic/ischemic tolerance in neuroprotection. Neurological Sciences, 46(11), 5671–5679. 40846814 10.1007/s10072-025-08413-2

[eph70462-bib-0050] Shin, H. Y. , Gerritsen, M. E. , & Bizios, R. (2002). Regulation of endothelial cell proliferation and apoptosis by cyclic pressure. Annals of Biomedical Engineering, 30(3), 297–304. 12051615 10.1114/1.1458595

[eph70462-bib-0051] Suzuki, K. , Yamada, H. , Fujii, R. , Munetsuna, E. , Ando, Y. , Ohashi, K. , Ishikawa, H. , Yamazaki, M. , Maeda, K. , Hashimoto, S. , & Hamajima, N. (2021). Association between circulating vascular‐related microRNAs and an increase in blood pressure: A 5‐year longitudinal population‐based study. Journal of Hypertension, 39(1), 84–89. 32740403 10.1097/HJH.0000000000002606

[eph70462-bib-0052] Tian, X. L. , Jiang, S. Y. , Zhang, X. L. , Yang, J. , Cui, J. H. , Liu, X. L. , Gong, K. R. , Yan, S. C. , Zhang, C. Y. , & Shao, G. (2019). Potassium bisperoxo (1,10‐phenanthroline) oxovanadate suppresses proliferation of hippocampal neuronal cell lines by increasing DNA methyltransferases. Neural Regeneration Research, 14(5), 826–833. 30688268 10.4103/1673-5374.249230 PMC6375031

[eph70462-bib-0053] Tsuboi, H. , Ando, J. , Korenaga, R. , Takada, Y. , & Kamiya, A. (1995). Flow stimulates ICAM‐1 expression time and shear stress dependently in cultured human endothelial cells. Biochemical and Biophysical Research Communications, 206(3), 988–996. 7832815 10.1006/bbrc.1995.1140

[eph70462-bib-0054] Tworkoski, E. , Glucksberg, M. R. , & Johnson, M. (2018). The effect of the rate of hydrostatic pressure depressurization on cells in culture. PLoS ONE, 13(1), e0189890. 29315329 10.1371/journal.pone.0189890 PMC5760025

[eph70462-bib-0055] Wang, K. K. , Yang, Z. , Zhu, T. , Shi, Y. , Rubenstein, R. , Tyndall, J. A. , & Manley, G. T. (2018). An update on diagnostic and prognostic biomarkers for traumatic brain injury. Expert Review of Molecular Diagnostics, 18(2), 165–180. 29338452 10.1080/14737159.2018.1428089 PMC6359936

[eph70462-bib-0056] Xiao, Z. H. , Wang, L. , Gan, P. , He, J. , Yan, B. C. , & Ding, L. D. (2020). Dynamic changes in miR‐126 expression in the hippocampus and penumbra following experimental transient global and focal cerebral ischemia‐reperfusion. Neurochemical Research, 45(5), 1107–1119. 32067150 10.1007/s11064-020-02986-4

[eph70462-bib-0057] Zhang, P. U. , Fu, G. , Xu, W. , Gong, K. , Zhao, Z. , Sun, K. , Zhang, C. , Han, R. , & Shao, G. (2024). Up‐regulation of miR‐126 via DNA methylation in hypoxia‐preconditioned endothelial cells may contribute to hypoxic tolerance of neuronal cells. Molecular Biology Reports, 51(1), 808. 39002003 10.1007/s11033-024-09774-1

[eph70462-bib-0058] Zhang, S. , Fu, W. , Jia, X. , Bade, R. , Liu, X. , Xie, Y. , Xie, W. , Jiang, S. , & Shao, G. (2023). Hypoxic preconditioning modulates BDNF and its signaling through DNA methylation to promote learning and memory in mice. ACS Chemical Neuroscience, 14(12), 2320–2332. 37289948 10.1021/acschemneuro.3c00069 PMC10289091

[eph70462-bib-0059] Zhou, J. , Lee, P.‐L. , Tsai, C.‐S. , Lee, C.‐I. , Yang, T.‐L. , Chuang, H.‐S. , Lin, W.‐W. , Lin, T.‐E. R. , Lim, S. H. , Wei, S.‐Y. I. , Chen, Y.‐L. , Chien, S. , & Chiu, J.‐J. (2012). Force‐specific activation of Smad1/5 regulates vascular endothelial cell cycle progression in response to disturbed flow. PNAS, 109(20), 7770–7775. 22550179 10.1073/pnas.1205476109 PMC3356658

[eph70462-bib-0060] Zhou, J. , Li, Y. I.‐S. , Nguyen, P. , Wang, K.‐C. , Weiss, A. , Kuo, Y. I.‐C. , Chiu, J.‐J. , Shyy, J. Y. , & Chien, S. (2013). Regulation of vascular smooth muscle cell turnover by endothelial cell‐secreted microRNA‐126: Role of shear stress. Circulation Research, 113(1), 40–51. 23603512 10.1161/CIRCRESAHA.113.280883 PMC3772783

[eph70462-bib-0061] Zhou, J. , Li, Y. S. , Wang, K. C. , & Chien, S. (2014). Epigenetic mechanism in regulation of endothelial function by disturbed flow: Induction of DNA hypermethylation by DNMT1. Cellular and Molecular Bioengineering, 7(2), 218–224. 24883126 10.1007/s12195-014-0325-z PMC4036703

[eph70462-bib-0062] Zhu, Y. , Li, X. , Lei, X. , Tang, L. , Wen, D. , Zeng, B. O. , Zhang, X. , Huang, Z. , & Guo, Z. (2025). The potential mechanism and clinical application value of remote ischemic conditioning in stroke. Neural Regeneration Research, 20(6), 1613–1627. 38845225 10.4103/NRR.NRR-D-23-01800 PMC11688546

